# The effect of foliar chitosan application on fusarium head blight in barley is highly genotype dependent

**DOI:** 10.1186/s12870-026-09486-7

**Published:** 2026-07-13

**Authors:** F. Hoheneder, S. Einspanier, O. Metzger, H. Klink, R. Stam, R. Hückelhoven

**Affiliations:** 1 Chair of Phytopathology, TUM School of Life Sciences, Technical University of Munich, Freising, Germany; 2 Institute of Phytopathology, Kiel University, Kiel, Germany

**Keywords:** Chitosan, Barley, Fusarium head blight, Induced resistance, Ethylene

## Abstract

**Background:**

Fusarium head blight (FHB) is a severe disease of barley that reduces yield and grain quality through contamination with mycotoxins. Conventional disease management provides only partial control under field conditions. Elicitors such as chitosan, a natural biopolymer, are promising alternatives to enhance plant resistance, but the efficacy of induced resistance may depend on host genotype and environmental conditions.

**Results:**

We investigated the effect of chitosan on FHB resistance in 39 diverse spring barley genotypes inoculated with *Fusarium culmorum*. Greenhouse experiments revealed reduced FHB severity and fungal DNA contents in several genotypes following chitosan pre-treatment and strong genotype-dependent effects. Field experiments at two locations indicated that chitosan treatment can reduce FHB severity under natural infection conditions, though the effects varied depending on genotype and environment. The cultivar Marthe consistently showed enhanced resistance across independent experiments. In addition, Chitosan pre-treatment induced ethylene production in barley leaves, suggesting activation of stress-related signalling pathways. However, ethylene release was attenuated after a second chitosan exposure, indicating a modulation of stress responses. Gene expression analysis of defence-related and *Fusarium*-responsive genes indicated a mitigated stress response in chitosan-induced barley. This may reflect reduced fungal success and suggests that chitosan induction of resistance might result in a physiologically cost-efficient defence mechanism in barley.

**Conclusions:**

Chitosan is a strong inducer of barley disease resistance with potential for integration into FHB management. Our results demonstrate that elicitor-induced resistance is a quantitative and genotype-dependent trait, and that cultivar-specific responses are likely transferable from controlled to field conditions. Identifying genotypes, such as Marthe, with consistent responsiveness to bioactive stimulants, offers breeding potential for optimised elicitor-induced resistance. This could support more sustainable FHB management strategies in barley in the future.

**Supplementary Information:**

The online version contains supplementary material available at 10.1186/s12870-026-09486-7.

## Introduction

Fusarium head blight (FHB) is caused by several *Fusarium* species and reduces yield and quality of harvested grain in barley. The abundance and severity of the disease highly depends on environmental and weather conditions around the most sensitive growth stage during anthesis [1]. Ear infections often result in contamination with mycotoxins like the trichothecene deoxynivalenol (DON) that are harmful to human and animal health. The control of FHB in barley mostly relies on indirect measures such as crop rotation, intensive soil management or the use of quantitatively resistant varieties [2]. However, breeding of FHB resistance in barley is challenging, because this trait often coincides with poor agronomic properties [3, 4]. Similarly, the efficient use of fungicides depends heavily on the optimal timing of application to protect the emerging spike until the end of flowering. In addition, there is a high risk of producing fungicide residues in the harvested crop or provoking fungicide resistance in *Fusarium* spp. populations [5]. As a consequence, there is an increasing demand for alternative eco-friendly control measures to extend and improve integrated pest management strategies [6].

The polysaccharide chitosan represents a promising elicitor for inducing defence responses and promoting growth and vigour in several plant species. Chitosan is derived from the deacetylation of chitin from crustacean exoskeletons or mushrooms and has been proven to be broadly effective in different hosts against various plant pathogens [7, 8]. Chitosan is mostly applied as foliar sprays, seed treatments or as a soil amendment. One advantage of chitosan and chitosan hydrochloride is the broad availability of its precursor chitin, which is approved as a basic substance for plant protection in the EU, thus overcoming an important hurdle for widespread practical applications in agri- and horticulture [9, 10].

Beyond directly limiting pathogen growth, chitosan can chelate nutrients to restrict pathogen access and, more important, activates multiple plant immune responses through pattern-triggered immunity. This includes the induction of phytoalexin and stress-related phytohormone biosynthesis, production of reactive oxygen species, expression of defence-related genes and stimulation of callose deposition. The antimicrobial and resistance-inducing activity of chitosan strongly depends on its structural properties, including degree of deacetylation, polymerization and molecular weight [7, 11, 12]. Additionally, chitosan has been shown to promote plant growth and abiotic stress tolerance [13], highlighting its potential for plant protection under unfavourable growth conditions.

Recent studies focused on the identification and evaluation of possible biological control measures in crops in general [14] and specifically against FHB in cereals [2, 15]. Chitosan has been shown to reduce disease symptoms and mycotoxin production in barley leaves challenged with *F. graminearum*, potentially through shifts in salicylic acid-related defence regulation or cellular detoxification processes [16]. Similar effects have been observed in wheat, where chitosan activates defence pathways and metabolic responses under controlled conditions [17].

Beyond directly inhibiting fungal proliferation, chitosan can alter fungal metabolism, including pathways associated with virulence factors, expression of cell wall-degrading enzymes and trichothecene biosynthesis in vitro. However, these effects depend on chitosan formulation and properties, particularly molecular weight and degree of deacetylation [18], resulting in variable antifungal effects [19].

Despite these promising mechanisms, the reported benefits of chitosan application in cereals remain inconsistent. Under controlled conditions, chitosan reduced FHB severity in one wheat cultivar, although this effect was not consistently reproduced across field seasons and resulted only in slight reductions in fungal DNA contents [20]. Similarly, field studies with three winter barley cultivars showed no effect on the foliar diseases powdery mildew (*Blumeria hordei*) and leaf scald (*Rhynchosporium commune*) [21].

Overall, while laboratory studies demonstrate promising chitosan-induced disease resistance, these approaches often fail under practical field conditions due to low effectiveness, high costs or sensitivity to environmental factors which are difficult to control [22].

To date, control of FHB in barley remains limited due to quantitatively resistant cultivars being susceptible to seasonal and local weather conditions [1, 23] and short fungicide application windows. Consequently, we propose the combined use of resistance-inducing elicitors and quantitatively resistant barley cultivars. However, to our knowledge, the variable response of different genotypes to elicitors has rarely been studied in larger crop assortments [24], particularly in the context of FHB in cereals.

We hypothesise that barley genotypes optimised for elicitor-induced resistance can efficiently reduce FHB disease severity which may be associated with a physiologically cost-efficient defence response characterised by specific defence gene regulation and stress signalling. To test this, we investigated the response of several spring barley cultivars and breeding lines to chitosan elicitation and their associated level of induced resistance against FHB in greenhouse and field inoculation trials.

## Material and methods

### Preparation of *F. culmorum* spray inoculum

The fungal *Fusarium culmorum* inoculum (isolates Fc002, Fc03, and Fc06; culture collection of the Chair of Phytopathology, Technical University of Munich) were selected for this study. These isolates were previously characterized as highly aggressive pathogen of barley spikes. They consistently produce strong symptoms and high levels of DON under both field and greenhouse conditions, making them a good system for studying barley FHB caused by DON-producing *Fusarium* strains [1, 25, 26]. The fungal isolates were cultured on ¼ PDA at 21 °C under white and UV light (16 h per day) as described by [25]. Fungal mycelia and spores were suspended and washed off with sterile distilled water and filtered through four layers of gauze bandage. The spore solution was prepared by combining equal amounts of spore solution per fungal isolate. The spore solution was diluted to a final spore concentration of 50,000 conidia mL^−1^ by the use of a haemocytometer. Finally, 1 mL L^−1^ Tween80® was added to obtain better wetting of the spore suspension on plant surfaces. A mock solution for spraying control plants was similarly prepared with sterile distilled water and Tween80®.

### Preparation of chitosan and mock solutions

Chitosan (CAS-No.: 9012–76-4) solutions were prepared using Chitosan powder obtained from shrimp shell with a proportion of minimum 75% deacetylation. In greenhouse and ethylene experiments, Chitosan (C3646, obtained from Sigma-Aldrich, Germany) and for field trials, “practical grade” Chitosan (417963, obtained from Sigma-Aldrich, Germany) was used. 500 mg L^−1^ Chitosan was dissolved in sterile distilled water acidified with 1 mL L^−1^ 100% acetic acid. Then, the solution was buffered with 5 M NaOH to pH = 6. Mock solutions were similarly prepared without Chitosan. Tween80® was subsequently added to the Chitosan or respective mock solutions to improve surface tension on plants. Dilutions were prepared by dissolving the Chitosan stock solutions in the mock solution.

The response of barley plants towards different Chitosan preparations (standard vs. practical grade) was previously tested in an ethylene assay, showing similar ethylene production of barley after chitosan elicitation (Suppl. Figure S-1). Hence, we therefore assumed that the different chitosan preparations produce comparable levels of induced resistance across different experimental setups and genotypes.

In addition, we performed an in vitro fungal growth assay to test the potential direct antifungal effects of different chitosan (C3646, obtained from Sigma-Aldrich, Germany) concentrations against the three *F. culmorum* isolates used. This revealed neutral to positive effects on fungal growth after at least 6 dpi. Therefore, images were taken and the growth area of individual isolates on agar plates was quantified using ImageJ [27] (Suppl. Figure S-2).

### Greenhouse experiment

39 spring barley (*Hordeum vulgare* L.) genotypes (cultivars and Bavarian breeding lines called IPZ_24727 or genotype 1–14) were cultivated under controlled glasshouse conditions at the Plant Technology Center of the Technical University of Munich. Seeds were sown into 3-L pots containing a peat-based substrate (Einheitserde ED73, Germany), with six seeds per pot. For each genotype and treatment condition, twelve pots were prepared to ensure sufficient biological replication. Plants were grown in a glasshouse with supplemental lighting to ensure a 16-h photoperiod throughout the experiment. Automatic daily watering was provided via flooding tables. To minimize positional effects, pots were re-randomized once a week. Temperature was controlled at 18 °C during the day and 16 °C at night, with relative humidity maintained at 60%. Two days before expected mid-anthesis (GS 65), the plants were either sprayed with a Chitosan or mock solution until run-off. Two days later, the flowering spikes were spray-inoculated with a *Fusarium culmorum* (Fc) spore or a mock solution until run-off. After spraying, the heads were covered and sealed with transparent polythene bags for two days to maintain humid conditions for optimal infection. The following treatment groups were tested: i: FHB + Chitosan (*F. culmorum* inoculation + chitosan), ii: FHB + Mock (*F. culmorum* inoculation + mock solution), iii: Control + Chitosan (no inoculation + Chitosan), iv: Control + Mock (no inoculation + mock solution). Infected and non-infected pots were spatially separated to prevent unintentional cross-contamination. Visual symptoms were quantified at different time points. Moreover, immature barley spikes were harvested to determine qPCR-based fungal biomass. For gene expression studies, immature barley spikes were sampled in liquid nitrogen.

### Preparation of *F. culmorum* bruised grain inoculum

To increase the disease pressure, a bruised-grain inoculum was prepared and applied to the field experiments. For this, the above-mentioned *F. culmorum* isolates were mixed with bruised and autoclaved wheat, barley and oat kernels as previously described by [1]. In short, 50 g of each bruised grain material was mixed with a malt extract solution (20 g L^−1^) in small autoclaving bags and autoclaved twice. Under sterile conditions, the fungal culture was cut out of the petri dish and mixed with the autoclaved bruised grain material. The grain inoculum was incubated under UV light at 16 °C for four weeks to maintain fungal growth on the bruised grain material. The bags were kneaded twice a week to mix the fungal mycelia. In both field locations, the same inoculum batch was used.

### Field trials and experimental design

Spring barley cultivars were grown at two field locations in southern (Roggenstein, Bavaria; ROG) and northern (Holtsee, Schleswig–Holstein; HOL) Germany during the 2024 season. Nine genotypes were sown at ROG and HOL (Accordine, Barke, Danielle, Ellinor, Grace, Marthe, breeding line genotype 13, Tasja, Vanille).

The field experiment in ROG was located at the experimental farm of the Technical University of Munich, while the experimental site at HOL was a commercial field managed under standard crop rotation (including winter oilseed rape and winter wheat). Both field locations received similar herbicide and fertilizer treatments, with no application of fungicides.

We recorded weather data (daily temperature and precipitation) from nearby stations in ‘Eichenau’ (ROG; 48°10′48.4″N, 11°19′13.2″E) and ‘Holtsee’ (HOL; 54°22′40.3″N, 9°52′17.4″E). Both sites had nearly similar annual temperature and precipitation, with heavy rainfalls during the time of inoculation at ROG (Table 1, Suppl. Figure S-3).

**Table 1:** Information about field locations, plant cultivation and overall weather conditions at both experimental sites during season in 2024

Location	Elevation	Sowing date	Mean precipitation	Mean temperature	Soil type	Previous crop	Harvest date
Holtsee	23 m	19 March	884 mm	8.6 °C	Silty loam	Maize	5 August
Roggenstein	520 m	20 March	912 mm	8.8 °C	Sandy loam	Wheat	25 July

At both sites, the main experimental area consisted of 160 plots arranged in 40 blocks. Each cultivar was sown in 1.5 m wide strips. The layout followed a pseudo-randomized block design: while blocks were arranged to account for field heterogeneity. Each block contained four treatment variants: i: FHB + Chitosan (*F. culmorum* inoculation + chitosan), ii: FHB + Mock (*F. culmorum* inoculation + mock solution), iii: Control + Chitosan (no inoculation + Chitosan), iv: Control + Mock (no inoculation + mock solution). Each treatment was applied once per block, with four replicate blocks per cultivar. *Fusarium* inoculation and chitosan treatments were applied twice during early stem elongation stage (GS 32–36) and shortly before flowering (GS 55–60). Each *Fusarium* treatment consisted of 75 g per square meter of *F. culmorum*-infected bruised grain material, spread evenly across within designated plots on the soil surface. To prevent cross contaminations between plots via rain splash dispersal of fungal spores, the outer edge of inoculated plots (approximately 25 cm) did not receive inoculum and acted as a mechanical barrier as described by [1].

Chitosan-treated plots received 150 mL m^−2^ of chitosan solution sprayed evenly until runoff; mock-treated plots received an equivalent volume of mock solution. At ROG, treatments were applied on 22 May (GS 32) and 13 June (GS 55) and at HOL on 24 May (GS 36) and 6 June (GS 61). The presence and severity of foliar diseases were assessed twice at each field site: ROG: 29 May (GS 37–49) and 19 June (GS 63–71); HOL: 4 June (GS 51–61) and 14 June (GS 61–65). For analysis of fungal DNA in grains, approximately 50 ears per plot were randomly harvested on 10 July at ROG (GS 90) and on 16 July at HOL (GS 89). Samples were dried at 50 °C for 24 h. After removing awns, grains were ground into fine powder and stored in 50 mL falcon tubes for further analyses.

### Plant material

A total of 39 different European spring barley cultivars and current breeding lines were used throughout the experiments in the laboratory, greenhouse and field trials. Breeding lines were provided under material transfer agreements by private breeding companies from southern Germany and anonymized (a list on the plant material is filed in the raw data set RD1 in the supplements).

### DNA and RNA extraction from immature spike tissue

Before DNA or RNA extractions, the immature spikes were ground to a fine powder in liquid nitrogen. Genomic DNA extractions were carried out on 250 mg of barley tissue following the protocol of [28], with minor modifications described by [26]. Briefly, pre-heated extraction buffer (TEN buffer, 2% SDS, 1 µl ml^−1^ 99% beta-mercaptoethanol) was mixed with sample material and incubated at 65 °C for 20 min. After centrifugation, the supernatant was then transferred to a new reaction tube, and an equal amount of cold 7.5 M ammonium acetate was added to precipitate proteins and cell contaminants. In the next step, the mixture was centrifuged and transferred to a new reaction tube. Subsequently, DNA-precipitation was performed using cold 99% isopropanol and incubation for 15 min. A DNA pellet was obtained from centrifugation at 13,000 rotations per minute for 15 min. The pellet was then washed twice in 70% Ethanol, dried in a vacuum dryer and dissolved in nuclease-free water overnight. After final centrifugation, the supernatant containing the extracted pure genomic DNA was quantified with a NanoDrop Spectrometer ND-1000 (Thermo Scientific, USA) and diluted with sterile nuclease-free water to a final concentration of 20 ng μl^−1^.

RNA extraction and purification were performed using 100 mg of ground barley spike tissue using the GeneMATRIX Universal RNA Purification Kit (EURx Ltd., Poland) with DNase I digestion according to the manufacturer’s protocol.

### DNA extraction from mature spike tissue

Mature grain samples (GS 99) were milled into a fine flour. The genomic DNA was extracted according to the European Community Reference Laboratories protocol for isolating maize DNA, as described in detail by [25]. Briefly, samples were incubated in preheated CTAB (Cetrimonium bromide) extraction buffer (65 °C), followed by chloroform-isoamyl alcohol (24:1) purification. DNA was precipitated using a CTAB precipitation buffer, washed with 70% ethanol, dried, and resuspended in sterile nuclease-free water. A final centrifugation step was used to remove residual polysaccharides.

### Quantification of barley and fungal DNA

The quality and yield of the extracted genomic DNA was assessed using a NanoDrop Spectrometer ND-1000 (Thermo Scientific, USA). Then the DNA was diluted with nuclease-free water to a final concentration of 20 ng μl^−1^. The amounts of *F. culmorum* and barley DNA in immature and mature head material was measured by qPCR using the protocol and respective species-specific primers as published by [29]. Pure fungal or barley DNA was diluted in a 10-fold DNA dilution series as internal standards, and nuclease-free water was used as the negative control (non-template control). Quantitative PCR reactions of each sample were conducted in technical replicates. Finally, the fungal DNA was normalized against barley DNA. Values are expressed as pg *F. culmorum* DNA per ng barley DNA.

### cDNA synthesis and RT-qPCR for gene expression analysis

500 ng of each extracted RNA sample was transcribed to complementary DNA (cDNA) using the Revert Aid cDNA synthesis Kit (Thermo Fisher Scientific; Lithuania) and random hexamer primers according to the manufacturer’s protocol. Briefly, the diluted samples were incubated together with random hexamer primer, 5 × Reaction Buffer, Ribo Lock RNase Inhibitor and Revert Aid Reverse Transcriptase and dNTPs at 25 °C for 10 min followed by 42 °C for 60 min. The reaction was terminated at 70 °C for 10 min. Each sample was diluted with sterile double-distilled H_2_O and stored at −20 °C. Gene expression analysis was conducted for the pathogenesis-related proteins *(PR 3* (Chitinase; [30]), *PR 5* (a thaumatin-like protein; [25]), a UDP-glycosyltransferase (*HvUGT14328* gene; [31]), a tryptophan decarboxylase [32] and Ubiquitin as a barley-specific housekeeping gene showing stable expression across variable conditions [33] (Table S-1). The RT-qPCR reactions were performed in technical replicates each containing 3 µl cDNA, 0.2 µl (100 pM) of the respective forward and reverse primer, 6.6 µl nuclease-free water and 10 µl “Takyon™ Low Rox SYBR® MasterMix dTTP blue” (Eurogentec, California, USA). The real-time PCR amplifications were performed using an AriaMx Real-time PCR system (Agilent Technologies Inc., Santa Clara, USA) using the following thermal cycling conditions: initial denaturation at 95 °C for 3 min, followed by 40 cycles of 95 °C for 30 s, 65 °C for 30 s, and 72 °C for 3 s. A final melt curve analysis was conducted by incubating at 95 °C for 30 s, followed by a stepwise temperature increase from 65 °C to 95 °C. The average CT-value of the reference gene was used to calculate the relative fold-change gene expression of the respective target genes. Therefore, the 2^–ΔΔCT^ method as described by [34] was performed. The relative expression values were calculated for each target gene in spike samples of the treatment groups “Mock + *Fc*” and “Chitosan + *Fc*” at 5 days post inoculation.

### Ethylene experiment

The ethylene (ET) production following chitosan treatment was measured to test elicitor-induced defence reactions and possible alterations in immune state. For this purpose, 20 seeds per genotype were grown in 1 L pots containing peat substrate (Einheitserde ED73, Germany) in a climate chamber (19 °C, 150 µmol m^−2^ s^−1^, 16 h per day, 60% relative air humidity) until full emergence of the second leaf (approx. 10 days after sowing). At this stage, the whole plants were treated with either 500 mg L^−1^ chitosan or the respective mock solution (preparation: see above) until run-off. After 48 h, individual primary leaves were harvested and four 4 mm leaf discs were punched out of every leaf. The leaf discs incubated overnight in sterile water for recovery from mechanical damage.

The next day, three leaf discs were placed in 6 ml glass vials prefilled with 250 µl sterile distilled water and then incubated with 250 µL elicitor suspension containing 100 mg L^−1^ Chitosan (C3646, Sigma-Aldrich, Germany) to obtain a final elicitor concentration of 50 mg L^−1^ Chitosan. The respective controls were elicited with a mock solution. The following treatment groups were tested: i: Mock (spray) + Mock (elicitation), ii: Mock (spray) + Chitosan (elicitation), iii: Chitosan (spray) + Mock (elicitation) and iv: Chitosan (spray) + Chitosan (elicitation).

The vials were closed with gas-tight rubber lids and incubated for 3.5 h at room temperature and daylight on a laboratory shaker (30 rpm). Subsequently, 1 ml of the gaseous phase was sampled from the headspace of each glass vial and injected into a gas chromatograph (Varian Aerograph 3300, Shimadzu; Japan) equipped with a deactivated aluminium oxide column. Peak areas were obtained by an integrator (C-R6A Chromatopac, Shimadzu, Japan) and divided by a calibration factor of 214 (1 ml pure ethylene calibration gas = 214 pmol at 1 bar) to derive the final ethylene amounts in pmol L^−1^ air [35]. The experiment was repeated in four independent biological replicates.

### Statistical analysis

The programming language R (V4.4.1) was used for statistical analysis and visualisation. The packages tidyverse, ggplot2, ggpubr and tidyr were used for basic data handling, analysis and visualization. For statistical analysis, we generated multiple mixed-effect models to reflect the experimental conditions [36, 37].

For the field data, the model included the variables Location, Cultivar, Inoculation, Treatment, and Date, as well as all their interaction terms as fixed effects. The blocks (repetitions) per Location and the plots (nested in block) were regarded as random factors. The residuals were assumed to be approximately normally distributed and to be heteroscedastic, and visually tested in a graphical residual analysis.

For each inoculation treatment, the model was defined as follows:

$$\begin{aligned}&{{\boldsymbol{Y}}}_{{\boldsymbol{i}}{\boldsymbol{j}}{\boldsymbol{k}}{\boldsymbol{l}}} = {\boldsymbol{\upmu}}+ {{\boldsymbol{C}}}_{{\boldsymbol{i}}} + {{\boldsymbol{L}}}_{{\boldsymbol{j}}} + {{\boldsymbol{T}}}_{{\boldsymbol{k}}}+{(CL)}_{ij} +{(CT)}_{ik}\\&+{(LT)}_{jk}+ {(CLT)}_{ijk}+{b}_{l}+{b}_{lk}+{\varepsilon}_{ijkl}\end{aligned}$$

where ${{\boldsymbol{Y}}}_{{\boldsymbol{i}}{\boldsymbol{j}}{\boldsymbol{k}}{\boldsymbol{l}}}$ is the *Fusarium* DNA content for the *i*^th^ Cultivar, *j*^th^ Location, *k*^th^ treatment, in the *l*^th^ block. μ: Overall mean, ${{\boldsymbol{C}}}_{{\boldsymbol{i}}}$: Effect of Cultivar_i_, ${{\boldsymbol{L}}}_{{\boldsymbol{j}}}$: Effect of Location_j_, ${{\boldsymbol{T}}}_{{\boldsymbol{k}}}$: Effect of Treatment_k_, ${(CL)}_{ij} +{(CT)}_{ik}+{(LT)}_{jk}+ {(CLT)}_{ijk}$ Interaction effects, ${b}_{l}+{b}_{lk}$ random effect of Block_l_ and of Treatment nested within Block (i.e., variation across treatment within each block), ${\varepsilon}_{ijkl}$ Residual error term (with heterogeneous variance by Location:Cultivar:Treatment). Based on this model, we calculated a pseudo R^2^ [38] and performed an analysis of variance (ANOVA). We conducted multiple contrast tests and extracted emmeans estimates and standard errors for visualization. Packages used for the analysis were agricolae (V1.3–7), car (V3.1–2), dplyr (V1.1.4), emmeans (V1.10.4), fmsb (V0.7.6), ggplot2 (V3.5.1), ggsignif (V0.6.4), MASS (V7.3–60.2), multcompView (V0.1–10), nlme (V3.1–166), piecewiseSEM (V2.3.0.1), readxl (V1.4.3) and tidyverse (V2.0.0).

## Results

### Chitosan pre-treatment alters ethylene production

**Fig. 1:**
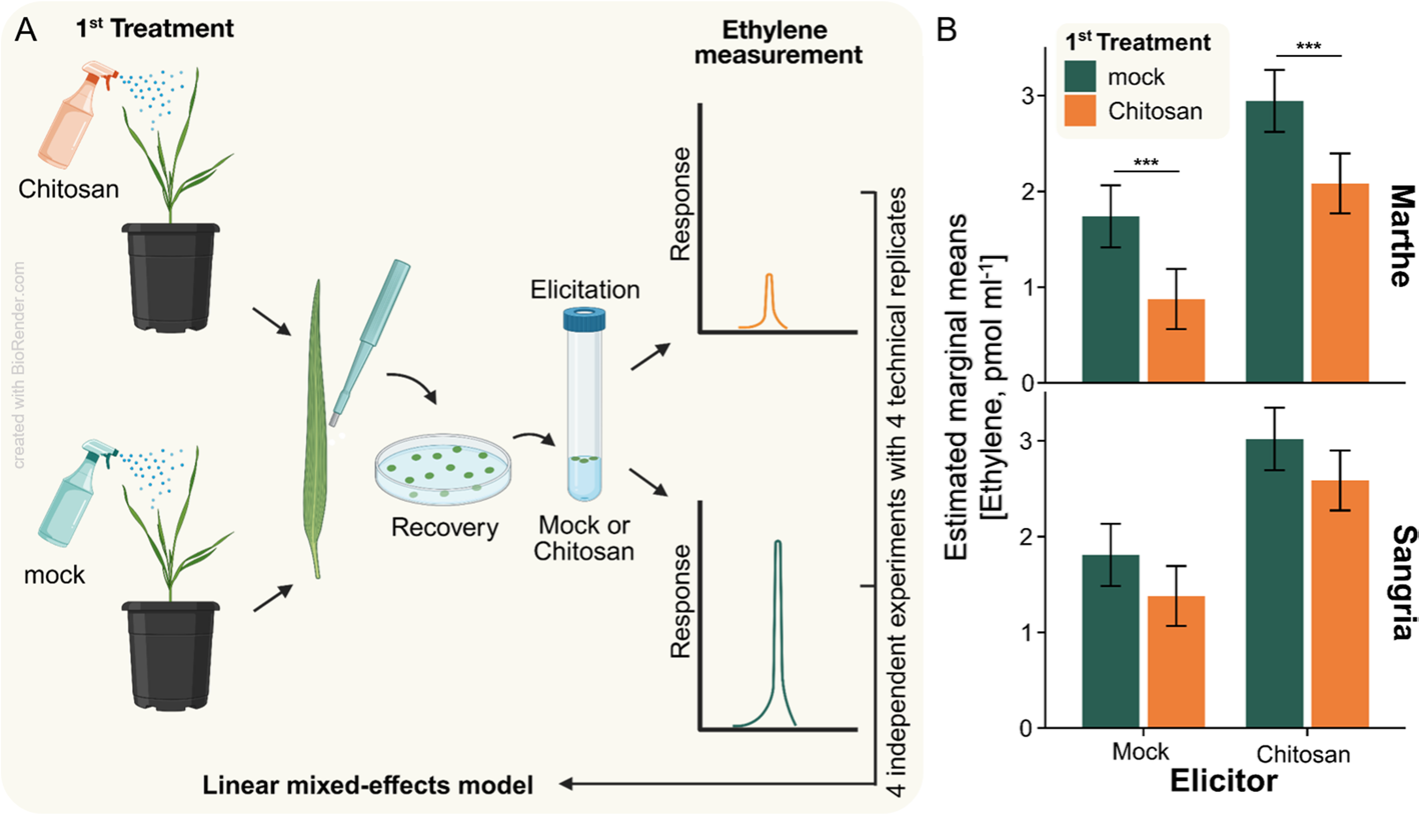
Experimental setup and estimated marginal means of ethylene production in mock- or chitosan-treated and chitosan-elicited barley leaves of cultivars Marthe and Sangria. **A** Ten-day-old barley plants were pre-treated (1^st^ treatment) with mock or chitosan solution 2 days before leaf discs were prepared for subsequent ethylene measurements following challenge treatment with mock or chitosan. The experiment was conducted in four biologically independent replicates with four to six technical replicates. The data sets from individual experiments were combined using a linear mixed-effects model to produce estimated marginal means. **B** shows the emmeans of ethylene releases in the four different treatment groups tested for two genotypes. Error bars indicate standard error. Significant differences are indicated as ***p < 0.001. Respective model estimates, ANOVA results and multiple pairwise comparisons are summarised in supplementary Datasets D1, D2 and D3. Figure **A** was created in BioRender. Hoheneder, F. (2026) https://BioRender.com/0ts19z5

The aim of this study is to characterise different barley genotypes on the chitosan-elicitor response and induced resistance against FHB on multiple scales. For this, we first conducted an ethylene release assay to validate the biological activity and genotype-specificity of the chitosan treatment. We first tested the influence of chitosan pre-treatment on the ethylene production after a subsequent 2nd chitosan elicitation. Ethylene production is a typical elicitor response and highly interlinked with the response to biotic stresses. We measured the release of ethylene in response to chitosan- or mock-elicitation in the chitosan or mock-pre-treated barley cultivars Marthe and Sangria. We analysed data from four independent experiments using a linear mixed effect model and calculated estimated marginal means (emmeans) (Fig. 1a).

We observed that chitosan-elicitation of both chitosan- and mock-pre-treated plants led to significantly elevated ethylene production. The cultivars Marthe and Sangria reacted very similarly to chitosan-elicitation if mock-pre-treated. This shows that chitosan generally can elicit ethylene production in barley. Surprisingly, the ethylene production in mock-pre-treated plants was higher than in chitosan-pre-treated plants. This effect was statistically significant for both mock- and chitosan-challenge as a second treatment in Marthe (Suppl. Dataset D3, Fig. 1b). Hence, the pre-treatment with chitosan rather led to a reduced than enhanced ethylene production in both genotypes.

### Induced resistance to FHB after chitosan pre-treatment is highly divergent across barley genotypes

Based on the observation that the barley genotypes Marthe and Sangria responded to chitosan with ethylene production, we investigated whether this chitosan-altered response on a molecular level translates into an altered resistance phenotype against FHB. We tested the phenotypic variability of chitosan-induced resistance in greenhouse infection conditions across a genotype assortment of 39 barley genotypes (cultivars and breeding lines called IPZ 24727 or genotype 1–14). We treated the plants with chitosan or mock solution two days before mid-flowering (GS 65). Subsequently, at mid-flowering, we inoculated the barley heads with either *F. culmorum* spore suspension or a mock solution. After 14 days, we quantified visible symptoms and relative fungal DNA contents to evaluate possible effects of a chitosan pre-treatment on FHB severity in barley.

**Fig. 2:**
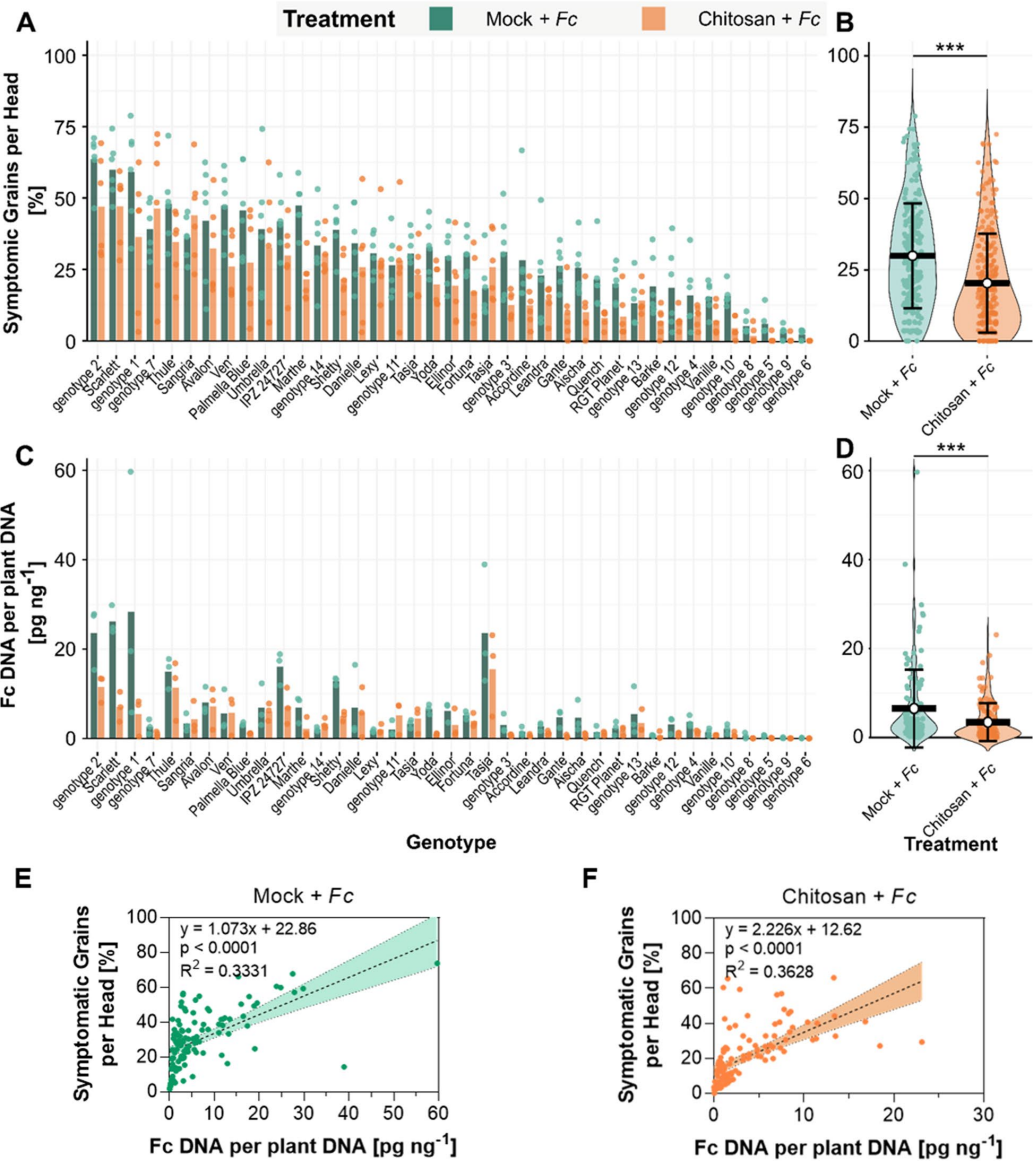
Mean percentage of spikelet symptoms and relative Fusarium DNA contents in 39 barley genotypes after mock- or chitosan-pre-treatment and *Fusarium culmorum* infection at 14 days post inoculation in the greenhouse. **A** Visually assessed proportions of symptomatic spikelets per head. **B** Mean proportion of spikelet symptoms across 39 genotypes. **C** *F. culmorum* DNA normalized to barley DNA detected in the previously assessed spikes. **D** Mean relative *F. culmorum* DNA contents across 39 genotypes. N = 3 biological replicates × 2 spikes per treatment group and genotype. Error bars indicate standard error. Significant differences are indicated as ***p < 0.001. **E**, **F** Scatter dot plots showing Pearson’s correlation between the proportion of symptomatic grains per head and the respective fungal DNA amounts in spikes of 39 genotypes, either mock-pre-treated or chitosan-pre-treated before infection. Respective ANOVA results and pairwise comparisons are summarised in supplementary Datasets D4, D5, D6 and D7

The overall severity of head infections (symptomatic grains and fungal DNA load) differed clearly among barley genotypes 14 days post inoculation, both in mock- and chitosan-pre-treated plants (Fig. 2). We observed the lowest mean incidence (mock + Fc; chitosan + Fc) in the genotype 6, 9, 5 and 8 (< 10%), indicating high quantitative FHB resistance. In contrast, we observed high susceptibility in the genotype 2, Scarlett, and genotype 7, with 39.1 to 63.7% symptomatic grains per spike (Fig. 2a).

Importantly, we observed overall fewer visibly infected spikelets per head in chitosan-pre-treated plants than in mock-pre-treated controls (Fig. 2a). Generally, most genotypes had less severe FHB symptoms after chitosan treatment (compare e.g. genotype 6, 9, 8 and 10, cultivars Barke, Accordine, Marthe). The genotype 13, 11 and 14 were exceptions, with no influence of chitosan on symptoms. Interestingly, we observed three genotypes with increased symptom severity after chitosan pre-treatment (genotype 3, genotype 1 and Sangria). The overall trends are supported by a meta-analysis using grain symptom data across all genotypes further showing a general reduction of FHB symptoms as a result of chitosan pre-treatment prior to infection (Fig. 2b). Overall, this indicates barley genotype-dependent chitosan-induced resistance to FHB symptoms in barley (for statistical analysis see suppl. Datasets D4 and D6).

To differentiate between reduced symptom expression and actual suppression of fungal development in chitosan-pre-treated plants, we quantified fungal DNA load relative to plant DNA via qPCR. Similar to the symptom data, we observed a strong differentiation within the genotype assortment (Fig. 2c). We measured low amounts of fungal DNA in the genotype 6, 9 and 8 and high quantities in genotype 2, Scarlett and genotype 7. Interestingly, we found that although highly susceptible, the genotypes 2, Scarlett and genotype 7 showed a strong reduction of fungal DNA contents when pre-treated with chitosan. The cultivars Marthe, Shetty and Yoda and breeding line IPZ 24727 similarly showed strongly induced resistance after chitosan pre-treatment. Here, the reduction in fungal DNA content exceeded 50%, indicating a strong resistance-inducing effect of chitosan in these genotypes.

Similar to our observations of symptoms, we identified some genotypes with a neutral or negative effect of chitosan pre-treatment. The spikes of Avalon, Ven, Danielle and Umbrella were rather equally colonised by *F. culmorum*, whereas Sangria, Tasja, genotypes 11 and 14 showed increased fungal DNA load in the chitosan-pre-treated samples (Fig. 2c). The meta-analysis across all genotypes again supported a general trend towards chitosan-induced resistance against *F. culmorum* development in barley heads (Fig. 2d).

Pearson´s regression analysis found a significant positive relationship between proportions of grain symptoms and respective fungal DNA contents across all genotypes for both the chitosan (p < 0.0001; R^2^ = 0.3628) and the mock-treated (p < 0.0001; R^2^ = 0.3331) spike samples (Fig. 2e, f). However, the high residual variance allows only cautious conclusions regarding a causal relationship. When considering single genotypes, we found poor relationships between the two datasets. For example, the genotype 3 had more grain symptoms per spike after a chitosan treatment compared to the mock-treated plants, while the opposite was found for DNA contents. Similarly, genotype 1 showed high proportions of grain symptoms, while DNA contents remained relatively low (Fig. 2a, b). Consequently, the visual assessments provided an inconsistent relationship with fungal DNA contents, particularly at the genotype level.

**Fig. 3:**
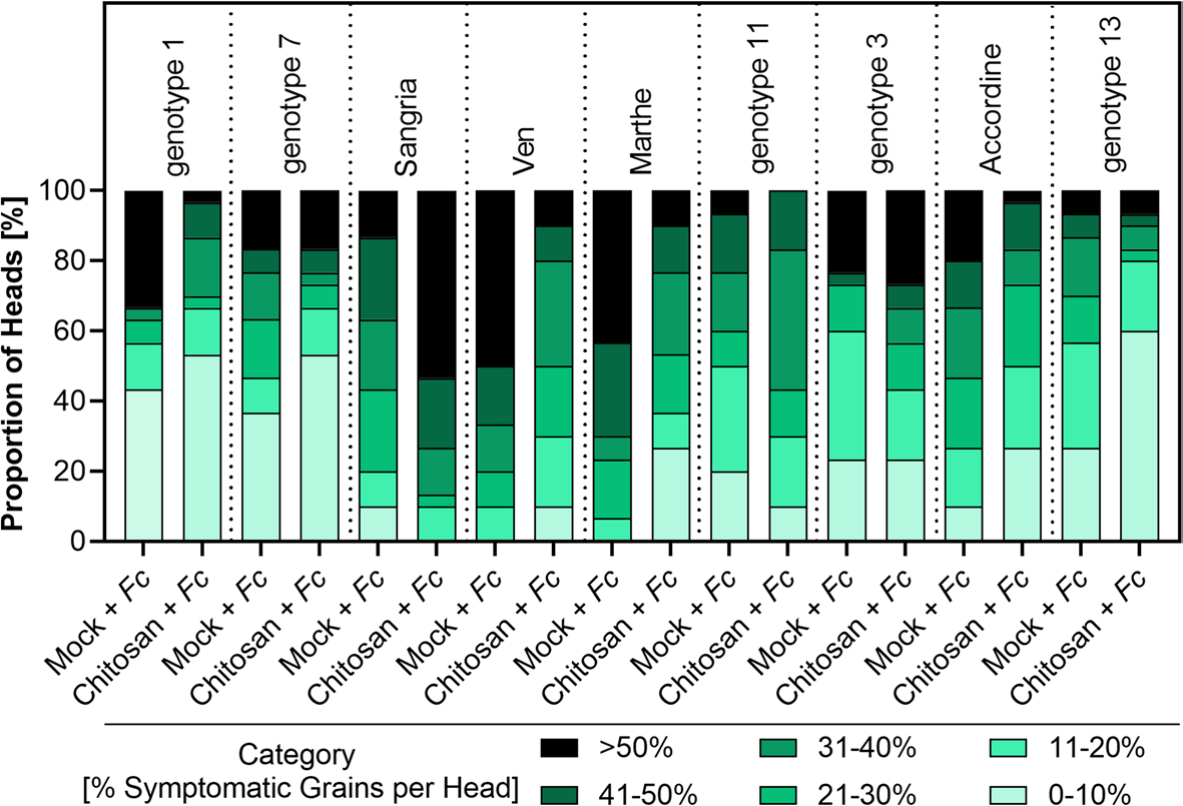
Proportions of barley heads showing grain symptoms after mock- or chitosan-treatment and *Fusarium culmorum* infection in nine selected barley genotypes. The graph shows the proportions of symptomatic grains per head of 30 individually assessed heads per genotype and treatment after 18 dpi in the first greenhouse experiment. The genotypes are sorted in a similar order as shown in Fig. 2

We independently assessed the spike symptoms at 18 dpi on up to 30 spikes per genotype and treatment to validate previous results. For this, we selected nine cultivars that had shown a positive, neutral or negative effect of chitosan pre-treatment on FHB severity before (Fig. 2). To better characterize the influence of chitosan on FHB severity, we categorised the proportions of symptomatic grains per spike into six disease categories (Fig. 3). Accordingly, we compared the FHB severity from slightly (0–10% symptomatic grains/spike) to very diseased (> 50%). The analysis revealed strong effects after chitosan pre-treatment across the genotypes. In Marthe, Accordine, genotype 11, genotype 1 and Ven, we observed a strong reduction of heavily infected spikes (> 50% grain symptoms per spike), while proportions of spikes with less severe symptoms (0–10%) increased after treatment on these genotypes. Interestingly, we measured a strong increase of highly symptomatic spikes (> 50%) after chitosan pre-treatment on the cultivar Sangria. This demonstrates an intensification of the negative effects of chitosan on the ear symptoms in this genotype, which were already evident in the data on 14 dpi (Fig. 2). The genotype 3, genotype 13 and genotype 7 did not show altered FHB susceptibility/resistance in the highest diseased category (> 50%) and only slight differences for the other categories (Fig. 3).

To further validate the initial greenhouse experiment results (Figs. 2a, b and 3), a subset of eleven genotypes, previously showing positive, neutral or negative effects of chitosan pre-treatment on FHB severity, were re-tested in an independent greenhouse experiment. We measured fungal DNA contents in spike samples harvested 5 dpi and scored the visual symptoms on 30 spikes per treatment and genotype after 18 dpi (Suppl. Fig. S-4). In this experiment, we predominantly observed reduced FHB after chitosan pre-treatment. In nine out of eleven genotypes, the fungal DNA content was reduced by partially more than 50% after the chitosan pre-treatment compared to the mock control. No differences in DNA content between treatments were detected only in the variety Lexy. The results on fungal colonisation of the heads corresponded with the visually assessed symptoms after 18 dpi. Here, we observed that genotypes with higher DNA contents in the mock-control showed larger proportions of heads with severe symptoms (41–50% symptomatic grains per head). We also observed an increased proportion of heads with healthy grains after elicitor pre-treatment, e.g. for the category of 0–10% symptomatic grains per head. Overall, the independent experiment confirmed the potential of chitosan-pre-treatment in controlling FHB for several barley genotypes whereas chitosan-induced susceptibility to FHB was less reproducible (compare Figs. 2, 3 and suppl. Fig. S-4).

### Pre-treatment with chitosan alleviates pathogen-stress-related gene expression

**Fig. 4:**
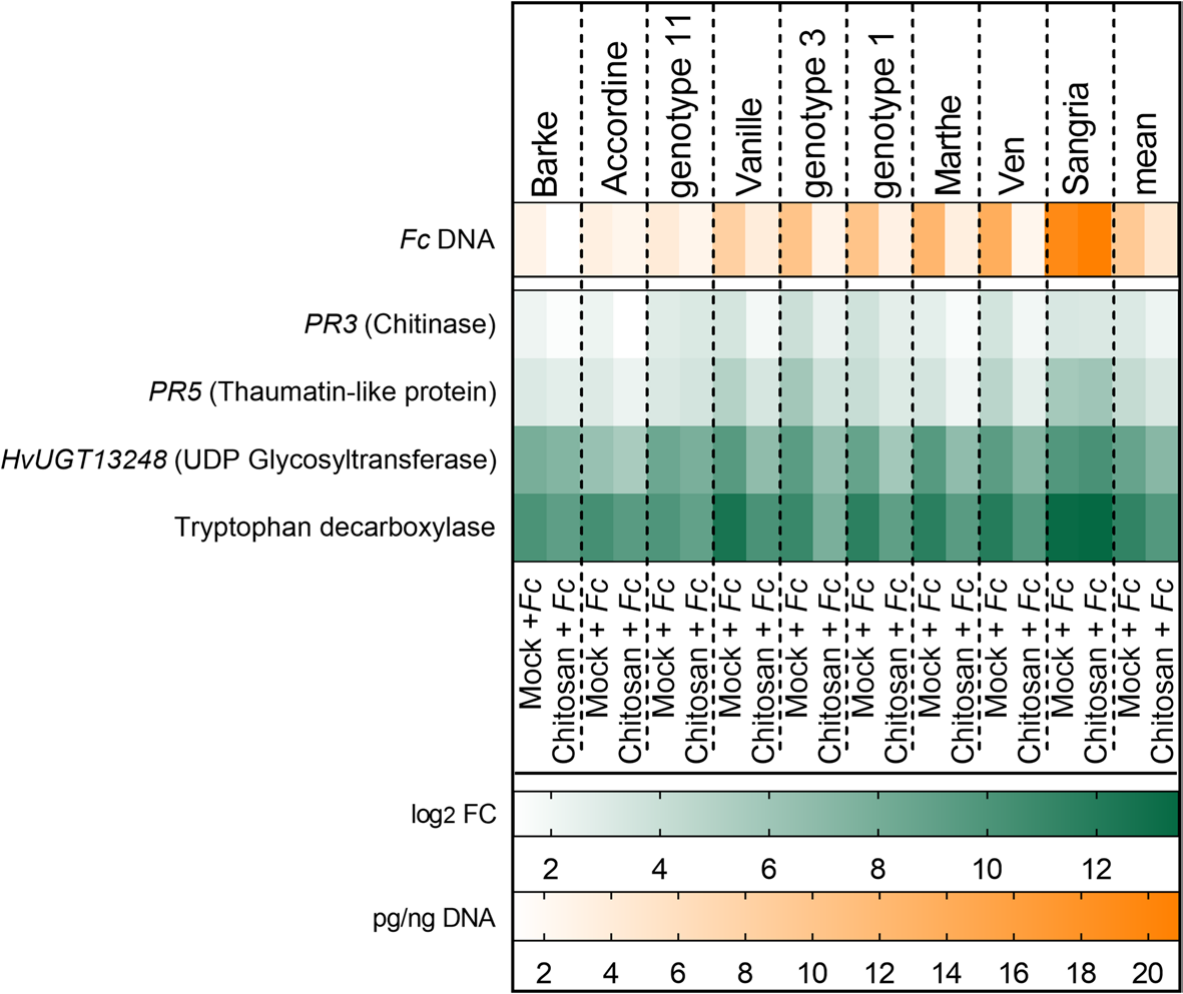
Fungal DNA contents and defence gene expression in mock- and chitosan-treated, *Fusarium culmorum* infected barley heads at 5 days post inoculation in the greenhouse. The nine genotypes are sorted by the amount of fungal DNA in ascending order and displayed in orange colour code. The respective log2 fold changes measured for four different defence-associated barley genes are displayed in green-shaded colour code. The mean DNA contents and gene expression levels across the nine genotypes are displayed on the right. The log2-transformed fold changes in gene expression in mock- or Chitosan-pre-treated and infected barley spikes were normalised to respective mock-treated and mock-sprayed samples. N = 3 biological replicates × 2 heads per treatment group and genotype

We conducted a gene expression analysis to link the treatment effect of chitosan application with gene regulatory responses. For this purpose, we measured the amount of fungal DNA and the expression of genes associated with pathogenesis- (*PR 3*, *PR 5*), *Fusarium* spp.- or deoxynivalenol-response (UDP glycosyltransferase *HvUGT13248*), and stress-related secondary metabolism (tryptophan decarboxylase) in infected ears after prior mock or chitosan treatment (for selected genes, compare [32]). We normalized the expression of the defence-associated genes to samples of the corresponding mock-treated and non-infected control plants (Fig. 4). We observed that eight of the nine genotypes were less severely infected after pre-treatment with chitosan, 5 days post inoculation. Barke showed the lowest disease severity in mock- and chitosan-treated samples upon infection, which was reflected in a generally reduced defence gene expression and only minor differences between the pre-treatments. In Sangria, we measured a generally high and slightly increased infection after the chitosan application, and correspondingly, a strong but little differential gene expression between the treatments. Generally, we observed that chitosan-pre-treated and infected cultivars tend to express defence-related genes less strongly than naïve plants. This may correspond to reduced fungal colonization in spike tissue (Fig. 4). Data showed a clear association between reduced stress gene expression and the reduced amount of fungal DNA. A Person´s regression analysis indicates positive relationships between the mean gene expression and the mean fungal DNA amounts in Mock and Chitosan pre-treated samples across all genotypes, except for the expression data of *PR* 3 (Table S-2). The defence gene expression upon infection was not increased at 5 dpi in the chitosan pre-treated compared to the mock-treated samples.

### Elicitor pre-treatment effects in field conditions

To test the performance of chitosan applications in field conditions, we conducted field trials with artificial *F. culmorum* inoculation at two different locations in Germany. Nine genotypes were grown in four replicated plots per treatment. We used a bruised grain *F. culmorum* inoculation on the soil surface to increase the natural pathogen pressure. We sprayed the plants at GS 32 and 55 with either mock or chitosan solution and analysed the severity of fungal infection (fungal DNA contents) in harvest samples. The data from the two field trials were summarised using a linear-mixed effects model.

We observed a strong differentiation of FHB infection across all genotypes, particularly in the mock-treated plots. Compared to field data from previous trials with artificial bruised-grain inoculation [1], we measured relatively low DNA contents at both locations, suggesting year-effect dependent low infection pressure. Despite high variance in the data possibly influenced by environmental conditions, we found by tendency a reduction in fungal DNA contents in five genotypes (Barke, Tasja, Grace, Ellinor, Marthe) as a result of chitosan treatment. Marthe was the most severely infected genotype (average of 1.2 pg fungal/ng barley DNA) in the mock-treated plots, whereas the DNA content was reduced in the chitosan-treated plots to 0.4 pg fungal per ng barley DNA. Conversely, the cultivars Vanille and Danielle showed a slight increase in infection levels in the chitosan-treated plots. The cultivar Accordine and the genotype 13 were the most resistant genotypes and showed no differences between the mock and the chitosan application. Overall, there was a trend for a genotype-dependent effect of chitosan-treatment on FHB in barley field trials, with rather the susceptible genotypes showing induced resistance.

## Discussion

Fusarium head blight (FHB) in barley reduces yield and grain quality through contamination with mycotoxins such as deoxynivalenol (DON), which can be strongly influenced by weather conditions around anthesis [1, 39]. Conventional disease management strategies, including crop rotation, resistant varieties, and fungicides provide only partial and inconsistent control with risk of fungicide residues in harvested crops or promoting fungicide resistance. We hypothesized that optimized genotype-specific responsiveness to chitosan improves induced resistance and could support integrated FHB management. To investigate this, we measured the stress-associated plant hormone ethylene as a qualitative marker of stress- and defence-signalling to assess responsiveness to chitosan and its modulation by prior exposure. Ethylene production is a central component of plant defence and stress mitigation [40], and is therefore suitable to capture early physiological responses to elicitation. Interestingly, the ethylene release assay showed that chitosan induces ethylene production in barley (Fig. 1). However, both tested genotypes produced less ethylene in chitosan pre-treated samples, indicating an altered state of physiological response, signal processing and modulations following prior elicitation. Variations in the strength of ethylene release between the genotypes might indicate possible differences in the level of chitosan-induced resistance. The production of ethylene starts with perception of an external stimulus via signal transduction and gene expression towards a stress or immune response [41], representing a relatively slow reaction when compared to e.g. production of reactive oxygen species. Thus, chitosan treatment ultimately attenuates the ethylene release in response to a second contact with chitosan. This observation indicates a possible desensitization or altered expression of ethylene responsive gene regulators and respective signalling following the first elicitation. This was obvious at three days post pre-treatment in our release assay (Fig. 1). Previous findings illustrate dynamic modulation of phytohormone production (like salicylic acid or jasmonic acid) by chitosan pre-treatment. Findings from [16] showed accumulation of salicylic acid three days after a chitosan treatment in barley leaves, demonstrating that hormonal regulation is affected within a time frame of days. In tomato plants infected with *Botrytis cinerea*, a prior chitosan treatment provoked a maintained callose deposition at the infection site and jasmonic acid accumulation two weeks after infection [42]. This is in line with the reduced FHB severity still measurable after 14 and 18 days post inoculation and 16 and 20 days post chitosan spraying (Figs. 2, 3; Suppl. Figure S-4). We speculate that chitosan-associated desensitization of ethylene production is likely durable over a longer period.

**Fig. 5:**
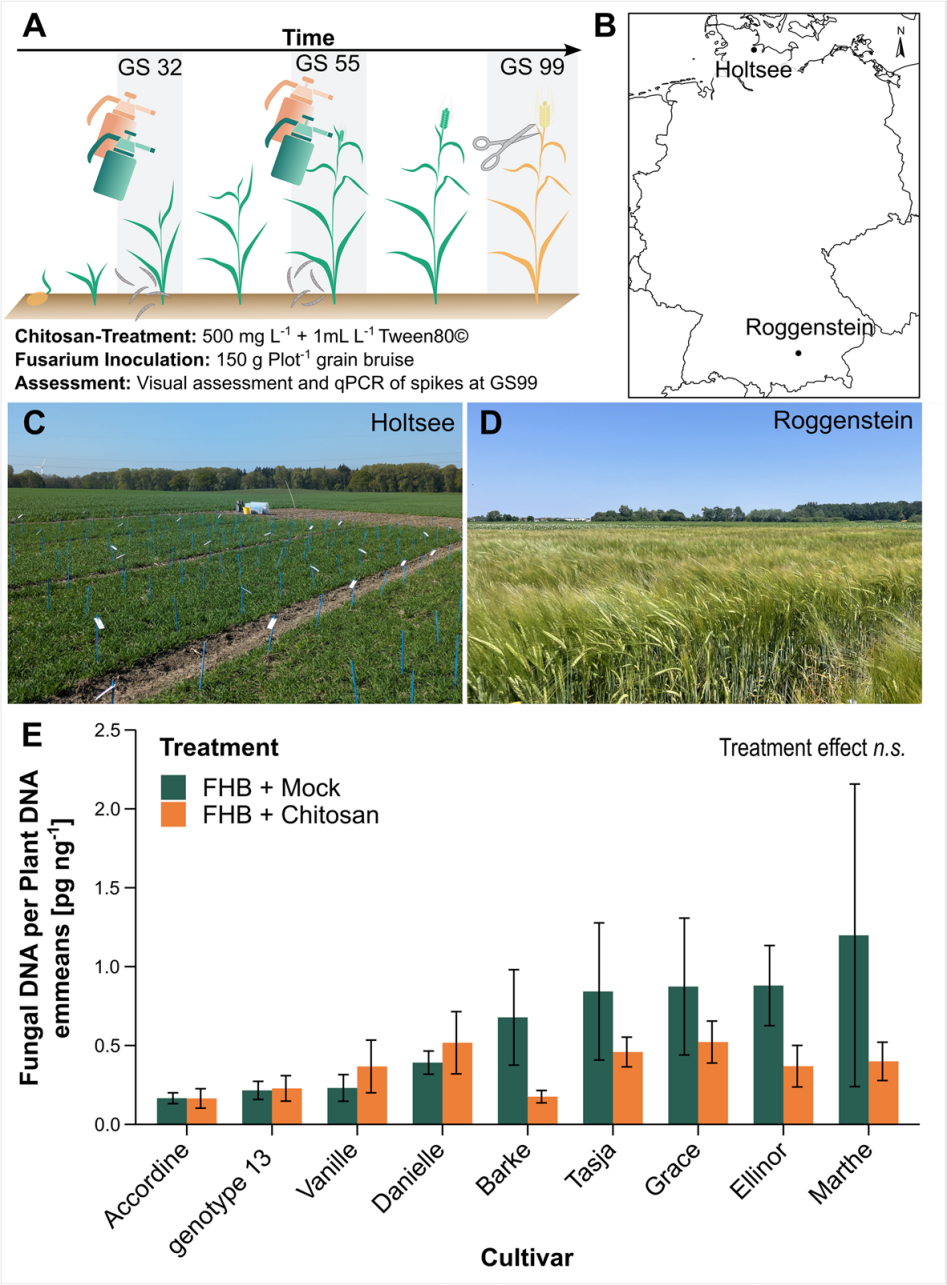
Estimated marginal means of fungal DNA contents in mock- and chitosan-treated and *Fusarium culmorum* infected heads of nine barley genotypes at two field locations in northern and southern Germany. The field plots were sprayed with mock- or chitosan solution at GS 32 and 55. Bruised-grain material colonised with *F. culmorum* isolates was distributed twice (at 32–36 and 55–60) on the soil surface for inoculation. Control plots remained untreated. N = 4 plots per treatment group, genotype and location. Error bars indicate the standard error of the mean. **A** shows the experimental setup with two inoculations and chitosan sprayings in the field. **B** shows the geographic location of the two field trials in Germany. Impressions from the field trials at Holtsee (**C**) and at Roggenstein (**D**). **E** shows estimated marginal means of fungal DNA contents in nine spring barley genotypes after mock- or chitosan-pre-treatment calculated from data across both field locations. Respective model estimates, ANOVA results and pairwise comparisons are summarised in supplementary Datasets D8, D9 and D10

Taken together, ethylene measurements suggest that chitosan may not only trigger immediate defence responses, but also modifies how plants respond to subsequent elicitation. Accordingly, chitosan-mediated effects might contribute to the observed reduction in FHB severity suggesting ethylene as a physiological marker reflecting stress responses in both naïve and elicitor-treated states. It is important to highlight that the ethylene response of both genotypes is highly dynamic and followed an inverse association with the degree of induced FHB resistance. We thus hypothesize that secondary processes such as signalling cascades, negative feedback loops and regulation of ethylene pathways might determine genotypic plasticity in elicitor response*.* Such markers are suitable to assess individual genotype-dependent chitosan-responses per se and in relation to a prior elicitation. We did not expect to observe reduced ethylene release after pre-treatment with chitosan. However, if we consider ethylene as a generic stress response marker, pre-treatment with chitosan might have induced physiological responses that attenuated stress upon a second exposure to the elicitor. Also, defence-related gene expression was attenuated after chitosan pre-treatment and FHB infection (Fig. 5). This may similarly reflect alleviation of plant stress during *Fusarium* spp. infection, or reduced fungal infection success (see below).

We observed generally reduced FHB severity in a large set of spring barley genotypes after chitosan treatment in greenhouse conditions. Despite a general positive relationship between symptoms and fungal DNA contents (Fig. 2e, f), symptoms did not fully reflect fungal DNA levels in some genotypes, indicating variable and genotype-dependent effects of elicitor-induced resistance on fungal growth and damage. Such discrepancies are commonly observed in barley [1, 24], where visual symptoms may underestimate fungal colonization compared to qPCR-based quantification. This may partly be explained by differences in symptom expression and fungal spread, including potential effects of chitosan on toxin production or plant stress responses. However, similar discrepancies were also observed in mock-treated plants, suggesting that this phenomenon is largely driven by genotype-specific basal resistance and tolerance mechanisms rather than elicitor effects alone. In this context, the strong differentiation of symptoms and fungal DNA contents between the genotypes reflects diversity in basal quantitative FHB resistance.

In the greenhouse, we identified several breeding lines and cultivars being more resistant subsequent chitosan pre-treatment (Fig. 2a, b). However, other genotypes (e.g. cultivar Lexy) did not show any phenotypic response or increased susceptibility after chitosan treatment. However, increased susceptibility e.g. in cultivars Tasja or Sangria was not always consistent between symptom data and fungal DNA contents (Fig. 2a, b) or in independent experiments (Figs. 2, 3; Suppl. Figure S-4). This suggests that induced resistance should always be considered in relation to each genotype’s level of basal defence [43]. Interestingly, we observed significant interaction terms for genotype and treatment for DNA contents (Suppl. Dataset D4), which is in line with a study from [44], who observed induced resistance to barley leaf scald after treatment with a combination of elicitors in a genotype-dependent manner under controlled and field conditions. Similarly [45], found variable induced resistance against *Phytophthora infestans* among six tomato genotypes with no clear dependence on the susceptibility level. However, only few studies described the genotype effect on induced resistance and often cover only a small number of genotypes [24, 44–46]. Our data provides clear evidence for a strong influence of the genotype on the efficacy of chitosan-induced resistance in barley. In this context a certain degree of genetic diversity for inducible resistance seems to exist in barley [24]. Our results suggest that inducible resistance is a quantitative trait. Consequently, genetic variance could be exploited in breeding programs to develop new cultivars with optimized elicitor-responsiveness as a breeding trait [47, 48]. However, this requires clear and measurable traits for phenotyping, which can be addressed through our approaches in the laboratory, greenhouse, and field.

A central limitation of studies on elicitor-induced resistance is insufficient translation into practical applications, thus being mostly descriptive and optimized for laboratory conditions. To make the first steps into filling this gap, we transferred our research into agricultural field conditions to critically evaluate the performance of elicitor-induced resistance. During the field trials in southern and northern Germany, we detected differences in FHB severity between treatments and genotypes (Fig. 5e). Although we observed high variance in the data, we still observed a positive influence of chitosan on FHB severity across several genotypes. High variance in our data may be attributed to environmental and weather effects across the two locations potentially affecting disease severity (Table 1, Suppl. Figure S-3). It remains unclear whether factors like soil type, day length or agronomic practices affect efficacy of induced resistance. We hypothesize that chitosan-induced resistance might still be sufficiently robust under variable weather conditions at the two different field locations. This highlights the potential of chitosan as a strong elicitor inducing robust resistance despite variable environmental conditions. We would like to stress, that the cultivar Marthe strongly profited in all our trials and in previous more preliminary studies from chitosan pre-treatment and reliably showed chitosan-induced FHB resistance. However, the beneficial effect of chitosan on some genotypes might be more elusive due to statistical reasons. Nevertheless, the field data still supported the results from the greenhouse for some genotypes. Based on the low severity levels in the field, we speculate that the beneficial effects of induced resistance might have rather been more detectable under higher disease pressure [49]. Nevertheless, field experiments appear to be indispensable for reliable selection of chitosan-responsive genotypes. Based on our experiences we propose that this requires precise phenotyping, and large genotype panels to capture the genotype-dependent plasticity in chitosan-induced shifts in FHB resistance [24], the level of basal resistance and the impact of the environment [50]. Additionally, abiotic stress stimuli can superimpose pathogen defence responses, if abiotic and biotic stress pathways compromise each other [51]. Consequently, the success of inducible resistance depends largely on the genotype and how it can still be elicited under variable environmental conditions [44, 50]. Therefore, the cultivar Marthe might be suitable as a future positive control for inducibility. It also represents an interesting parent for crossings to obtain new breeding lines that combine a higher basal resistance and appropriate beneficial elicitor responsiveness in the field.

Ideally, field experiments should take place under various field conditions to segregate location effects from biological signal. Our data base on trials at two locations, but would profit, on the one hand, from more diverse environments, and on the other hand, from technical optimization (see below) to balance the impact of the environment. Here, we used sophisticated statistical tools like estimated marginal means derived from a linear mixed-effects model to properly control for variation in the data and to test for chitosan effects. However, more repetitions with variable inoculation pressures possibly could confirm genotypes that show strong induced resistance effects, even under very high disease pressure in the greenhouse (Fig. 2, Fig. S-4). Significant research effort is needed to optimize dosage, formulation and timing of chitosan spray application to ensure universal elicitor-induced resistance success independent of environmental conditions [18, 22, 52].

Chitosan represents a promising elicitor for the induction of plant defence responses, and it triggers a wide range of local and systemic physiological reactions [16, 19, 53]. Our data demonstrate a clear protection mechanism in the spike, leading to reduced infection success of *Fusarium* spp. However, in contrast to previous results with diverse fungi [11], we found no antifungal and rather growth-promoting effects of chitosan on our *F. culmorum* isolates in vitro (Suppl. Figure S-2). Our gene expression analyses of defence-related genes indicated that their gene expression reflects fungal colonisation and, consequently, the degree of infection success rather than the level of induced resistance (Fig. 4, Table S-2). This is in line with earlier results showing that defence gene expression during barley FHB pathogenesis corresponds to the level of *F. culmorum* infection [32]. Possibly, if the defence response is highly efficient when activated locally and at an early stage, this results in reduced stress and lower expression of pathogenesis-related genes across the entire spike at 5 dpi. In addition, lignification in response to chitosan has previously been demonstrated in wheat seedlings through the accumulation of phenolic compounds [54, 55]. In this context, strengthening of the cell walls may reduce fungal penetration success and physically protect tissues from spreading infection. This would minimise the need for costly systemic up-regulation of defence-related genes. Such early and localised defence activation may thus hamper further fungal spread and explain the pronounced effects of chitosan. In our study, we treated spikes two days prior to inoculation, and we measured gene expression relatively late at 5 dpi. This attenuated defence gene expression may thus indicate that the plants were sufficiently early in an induced state to mitigate FHB stress, both at the infection and the gene expression level. This is underlined by reduced expression of the deoxynivalenol-response gene UDP-glycosyltransferase *HvUGT13248*. This would render chitosan-mediated resistance very cost-effective. Supporting this hypothesis, our ethylene release assay revealed that chitosan pre-treatment reduced stress when subsequently challenged with a chitosan-elicitor, representing the presence of a fungal pathogen. Overall, a generally lower stress level may positively modulate early defence in a physiologically more cost-efficient manner with minimal damage to the plant itself. The differences in ethylene responses between the two representative genotypes Marthe and Sangria upon repeated elicitation might indicate that downstream signalling recovery or negative feedback regulation of the ethylene pathway differ between genotypes. Although Marthe and Sangria exhibited similar ethylene release patterns after chitosan exposure, their contrasting FHB resistance responses suggest that ethylene production alone does not directly reflect resistance strength in this system. In this context, the attenuated ethylene responses after chitosan pre-treatment represent a qualitative stress marker reflecting induced physiological alterations after chitosan perception. However, it remains unclear whether absolute ethylene levels or their relative changes can be associated with induced resistance, and this requires further investigation. In addition, early activation of mycotoxin detoxification and stress-mitigating enzymes, such as those that counteract reactive oxygen species generated under pathogen stress, may reduce energy costs by limiting fungal growth and pathogen-caused cellular damage during FHB development. This suggests studying the global regulation of stress-mitigation genes by RNA sequencing at earlier time points after chitosan treatment and infection for deeper characterisation of the genotype-dependent physiological properties and trade-offs in induced resistance. In this context, it is interesting that a recent study with durum wheat shows enriched biological functions for antioxidants and detoxification in global gene expression patterns during chitosan-induced FHB resistance [17].

## Conclusions

Chitosan is a strong inducer of plant defence, which possesses high potential for pathogen control in crops. However, the transfer from controlled conditions to field application is challenging, likely due to genotype x environment interactions. Large-scale characterization of physiological and phenotypic parameters across large genotype panels might be a promising strategy to resolve this knowledge-gap. Our results show a high genotype-dependent variation of spring barley in chitosan-induced resistance against FHB. This indicates that some chitosan-treated barley genotypes show induced physiological response and reduced susceptibility when later pathogen-challenged. This supports that a chitosan-induction is probably physiologically cost-efficient for the plant. We screened 39 genotypes that showed differentiation of induced resistance in the greenhouse under high disease pressure. Importantly, we could further transfer the obtained results to the field, where we again obtained chitosan-induced defence against FHB under practical farming conditions. The strong variety-dependent response to chitosan offers potential for breeding cultivars that are optimised for the application of elicitor-induced resistance to FHB in integrated disease management.

## Supplementary Information

Supplementary Material 1: Table S-1. Primer sequences for quantification of barley or *F. culmorum* DNA in qPCR assay [29] and barley reference and target genes used for gene expression analyses in qRT-PCR. The barley Ubiquitin gene was previously validated as a reference gene in a study by [33] and showed stable expression in transcriptomic data across different stress conditions [32]. The gene identifiers and gene descriptions are given for the Morex v3 barley reference genome [56]. Table S-2. Pearson´s correlation analyses assessing relationships between mean gene expression of defence- and stress associated genes with mean fungal DNA contents in barley spikes of nine either Mock + *Fc* or Chitosan + *Fc* treated barley genotypes 5 days post inoculation. The table shows the comparisons per target gene, the respective number of genotypes (n), the degree of freedom (df) per comparison, the p value at a level of significance of 0.05, R^2^ values and the equation for the linear regression line. Figure S-1. ethylene production in leaves of cultivars Marthe and Sangria treated with mock or different chitosan preparations. [A] The pre-treatment (1st) of 10 days old barley plants with mock- or chitosan solution occurred 2 days before the preparation of leaf discs for subsequent ethylene measurement after challenge treatment with mock or chitosan. The experiment was conducted in two independent biological replicates with four to five technical replicates. Error bars indicate standard error. “Chitosan” = Chitosan (C3646, obtained from Sigma-Aldrich, Germany); “Chitosan practical grade” (417963, obtained from Sigma-Aldrich, Germany); “Chitosan low mol. Weight” = Chitosan (448869, obtained from Sigma-Aldrich, Germany). Statistical differences between rank sums of treatment groups were tested using the Mann–Whitney U-test. Significant differences are indicated as *p < 0.05; **p < 0.01. Figure S-2. Fungal growth assay on ¼ strength PDA supplemented with different chitosan concentrations. [A] Growth area of *F. culmorum* measured at 4 dpi. [B] Growth area of *F. culmorum* measured at 6 dpi. [C] Exemplary images of agar plates inoculated with isolate Fc002 at 4 dpi. [D] Exemplary images of agar plates inoculated with isolate Fc002 at 6 dpi. The fungal growth assay was conducted for three different *F. culmorum* isolates (Fc002, Fc03, Fc06) on ¼ strength PDA supplemented with 0.0, 0.1, 0.5 and 1.0 g L^−1^ chitosan (C3646, obtained from Sigma-Aldrich, Germany). The dot plots show mean area with fungal growth of three different agar plates per fungal isolate and chitosan concentration measured with the software ImageJ. Error bars indicate the standard error. Figure S-3. Temperature and precipitation data during the field trials at two locations in northern and southern Germany. Daily mean (tavg), minimum (tmin), and maximum (tmax) temperatures, as well as daily precipitation sums (prcp), are shown for the period between sowing and harvest at Holtsee [A] and Roggenstein [B]. Arrows indicate the dates of the first and second chitosan treatments, grain sampling, and final harvest. Figure S-4. Fungal DNA contents and proportions of barley heads showing grain symptoms after mock- or chitosan-treatment and *Fusarium culmorum* infection in 11 selected barley genotypes. [A] shows the mean *F. culmorum* DNA detected in barley heads at 5 days post inoculation. [B] displays the proportions of symptomatic grains per head are sorted by disease categories, reflecting the disease severity of individual spikes. For quantification of fungal DNA, 3 × 2 heads per sample and genotype were harvested. Grain symptoms were assessed on 30 heads per treatment group and genotype. The genotypes are sorted in the same order from left to right as displayed in Fig. 2. Error bars show standard error.

## Data Availability

The raw data (RD1 – RD10) and supplementary datasets (D1 – D10) generated in this study as well as the code used in this is publicly available at https://github.com/PHYTOPatCAU/Chitosan/_Priming
