# Supplementary material for: The effect of foliar chitosan application on fusarium head blight in barley is highly genotype dependent
Source: BMC Plant Biol. 2026 Jul 13;26:1199. doi: 10.1186/s12870-026-09486-7 (PMC13366867; doi:10.1186/s12870-026-09486-7)
Supplement: Supplementary file 1 — Supplementary Material 1: Table S-1. Primer sequences for quantification of barley or F. culmorum DNA in qPCR assay [29] and barley reference and target genes used for gene expression analyses in qRT-PCR. The barley Ubiquitin gene was previously validated as a reference gene in a study by [33] and showed stable expression in transcriptomic data across different stress conditions [32]. The gene identifiers and gene descriptions are given for the Morex v3 barley reference genome [56]. Table S-2. Pearson´s correlation analyses assessing relationships between mean gene expression of defence- and stress associated genes with mean fungal DNA contents in barley spikes of nine either Mock + Fc or Chitosan + Fc treated barley genotypes 5 days post inoculation. The table shows the comparisons per target gene, the respective number of genotypes (n), the degree of freedom (df) per comparison, the p value at a level of significance of 0.05, R2 values and the equation for the linear regression line. Figure S-1. ethylene production in leaves of cultivars Marthe and Sangria treated with mock or different chitosan preparations. [A] The pre-treatment (1st) of 10 days old barley plants with mock- or chitosan solution occurred 2 days before the preparation of leaf discs for subsequent ethylene measurement after challenge treatment with mock or chitosan. The experiment was conducted in two independent biological replicates with four to five technical replicates. Error bars indicate standard error. “Chitosan” = Chitosan (C3646, obtained from Sigma-Aldrich, Germany); “Chitosan practical grade” (417963, obtained from Sigma-Aldrich, Germany); “Chitosan low mol. Weight” = Chitosan (448869, obtained from Sigma-Aldrich, Germany). Statistical differences between rank sums of treatment groups were tested using the Mann–Whitney U-test. Significant differences are indicated as *p < 0.05; **p < 0.01. Figure S-2. Fungal growth assay on ¼ strength PDA supplemented with different chitosan concentratio [file 12870_2026_9486_MOESM1_ESM.zip › Supplementary Data.docx]

# Supplementary Data

**Table S-1:** **Primer sequences for quantification of barley or *F. culmorum* DNA in qPCR assay [29] and barley reference and target genes used for gene expression analyses in qRT-PCR.** The barley Ubiquitin gene was previously validated as a reference gene in a study by [33] and showed stable expression in transcriptomic data across different stress conditions [32]. The gene identifiers and gene descriptions are given for the Morex v3 barley reference genome [56].

| **Gene identifier** | **Gene description** |  |  | **Oligo-Sequence (5’ – 3’)** |
| --- | --- | --- | --- | --- |
|  | Barley elongation factor 1 alpha | **F**  **R** |  | TCTCTGGGTTTGAGGGTGAC  GGCCCTTGTACCAGTCAAGGT |
|  | *F. culmorum* elongation  factor 1 alpha | **F**  **R** |  | CACCGTCATTGGTATGTTGTCACT  CGGGAGCGTCTGATAGTCG |
| HORVU.MOREX.r3.1HG0024910 | Ubiquitin | **F**  **R** |  | AGACCATCACGCTGGAGGTG  GTCGGCGTTGGGGCACTCCTT |
| HORVU.MOREX.r3.7HG0721890 | Tryptophan decarboxylase | **F**  **R** |  | TTCTTCCCCTCCACCAACAG  ATGAAGGTGGTGGGTAGACG |
| HORVU.MOREX.r3.5HG0464880 | UDP-Glycosyltransferase *HvUGT13248* | **F** |  | CATCTGTTGTGCTCGTGTCC |
|  |  | **R** |  | AGCCCTATGTGCAAGAACCT |
| HORVU.MOREX.r3.1HG0054950 | Chitinase II (*PR3*) | **F** |  | CTACACGTACGACGCCTTCAT |
|  |  | **R** |  | GTGGCCTTGCTTATCTCTTCC |
| HORVU.MOREX.r3.7HG0752030 | Thaumatin-like protein (*PR5*) | **F**  **R** |  | CGGCGGTGCTCTTCC  GGCGGGCACGTTGAT |

**Table S-2:** **Pearson´s correlation analyses assessing relationships between mean gene expression of defence- and stress associated genes with mean fungal DNA contents in barley spikes of nine either Mock + *Fc* or Chitosan + *Fc* treated barley genotypes 5 days post inoculation.** The table shows the comparisons per target gene, the respective number of genotypes (n), the degree of freedom (df) per comparison, the p value at a level of significance of 0.05, R^2^ values and the equation for the linear regression line.

| **Comparison (mean gene expression *vs.* mean DNA content)** | **n** | **df** | **p value** | **R^2^** | **Equation** |
| --- | --- | --- | --- | --- | --- |
| *PR3* (log2FC) vs. fungal DNA content (Mock + *Fc*) | 9 | 7 | 0.1246 | 0.3030 | y = 0.06372x + 2.430 |
| *PR3* (log2FC) vs. fungal DNA content (Chitosan + *Fc*) | 9 | 7 | 0.1286 | 0.2977 | y = 0.06030x + 1.864 |
| *PR5* (log2FC) vs. fungal DNA content (Mock + *Fc*) | 9 | 7 | 0.0263 | 0.5295 | y = 0.1515x + 2.758 |
| *PR5* (log2FC) vs. fungal DNA content (Chitosan + *Fc*) | 9 | 7 | 0.0014 | 0.7897 | y = 0.1676x + 2.414 |
| UDP Glycosyltransferase HvUGT13248 (log2FC) vs. fungal DNA content (Mock + *Fc*) | 9 | 7 | 0.0174 | 0.5780 | y = 0.1536x + 7.309 |
| UDP Glycosyltransferase *HvUGT13248* (log2FC) vs. fungal DNA content (Chitosan + *Fc*) | 9 | 7 | 0.0072 | 0.6672 | y = 0.1830x + 6.217 |
| Tryptophan decarboxylase (log2FC) vs. fungal DNA content (Mock + *Fc*) | 9 | 7 | 0.0024 | 0.7548 | y = 0.1847x + 9.623 |
| Tryptophan decarboxylase (log2FC) vs. fungal DNA content (Chitosan + *Fc*) | 9 | 7 | 0.0005 | 0.8406 | y = 0.2356x + 8.549 |


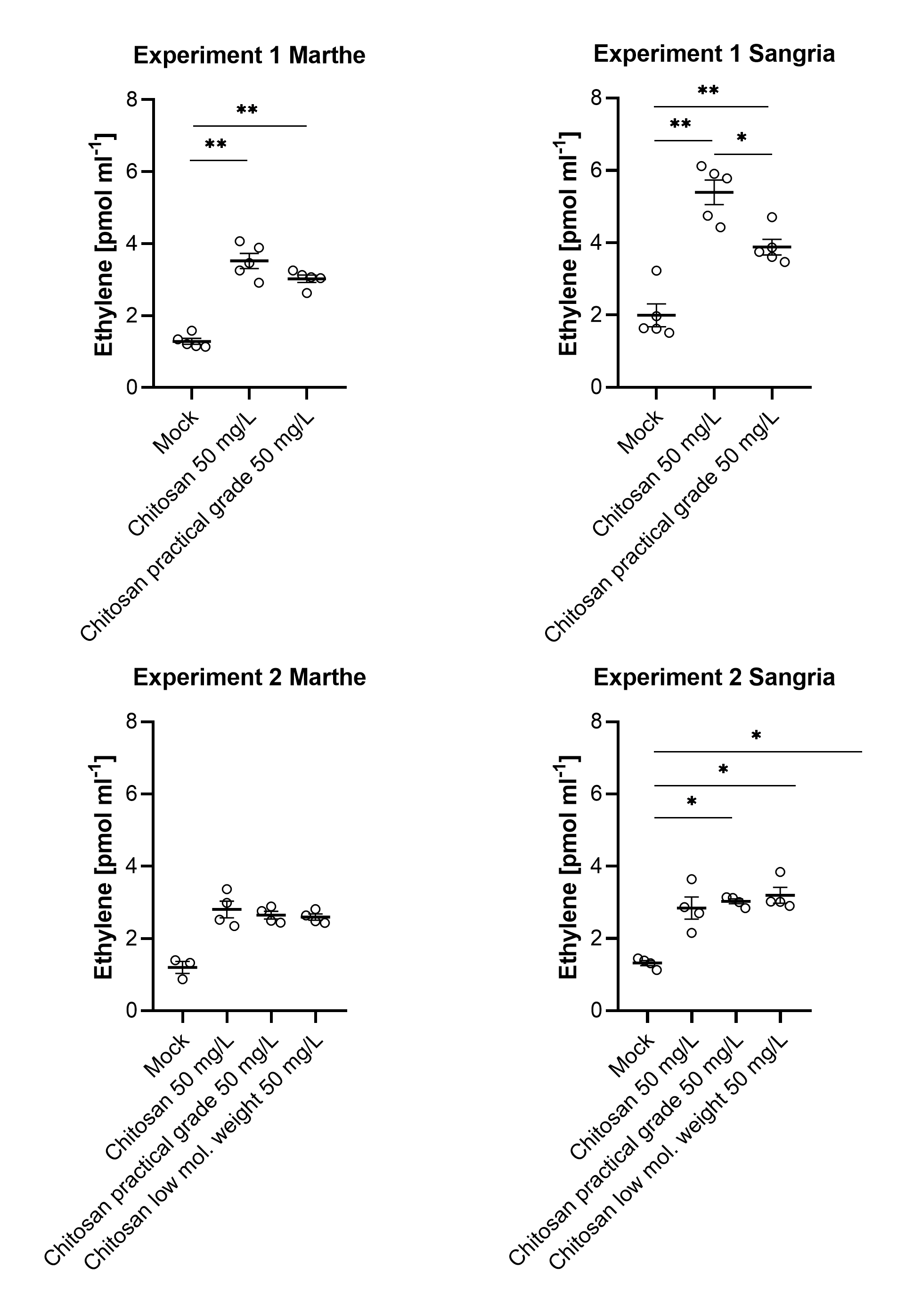


**Figure S-1: ethylene production in leaves of cultivars Marthe and Sangria treated with mock or different chitosan preparations.** [A] The pre-treatment (1^st^) of 10 days old barley plants with mock- or chitosan solution occurred 2 days before the preparation of leaf discs for subsequent ethylene measurement after challenge treatment with mock or chitosan. The experiment was conducted in two independent biological replicates with four to five technical replicates. Error bars indicate standard error. “Chitosan” = Chitosan (C3646, obtained from Sigma-Aldrich, Germany); “Chitosan practical grade” (417963, obtained from Sigma-Aldrich, Germany); “Chitosan low mol. Weight” = Chitosan (448869, obtained from Sigma-Aldrich, Germany). Statistical differences between rank sums of treatment groups were tested using the Mann-Whitney U-test. Significant differences are indicated as *p < 0.05; **p < 0.01.


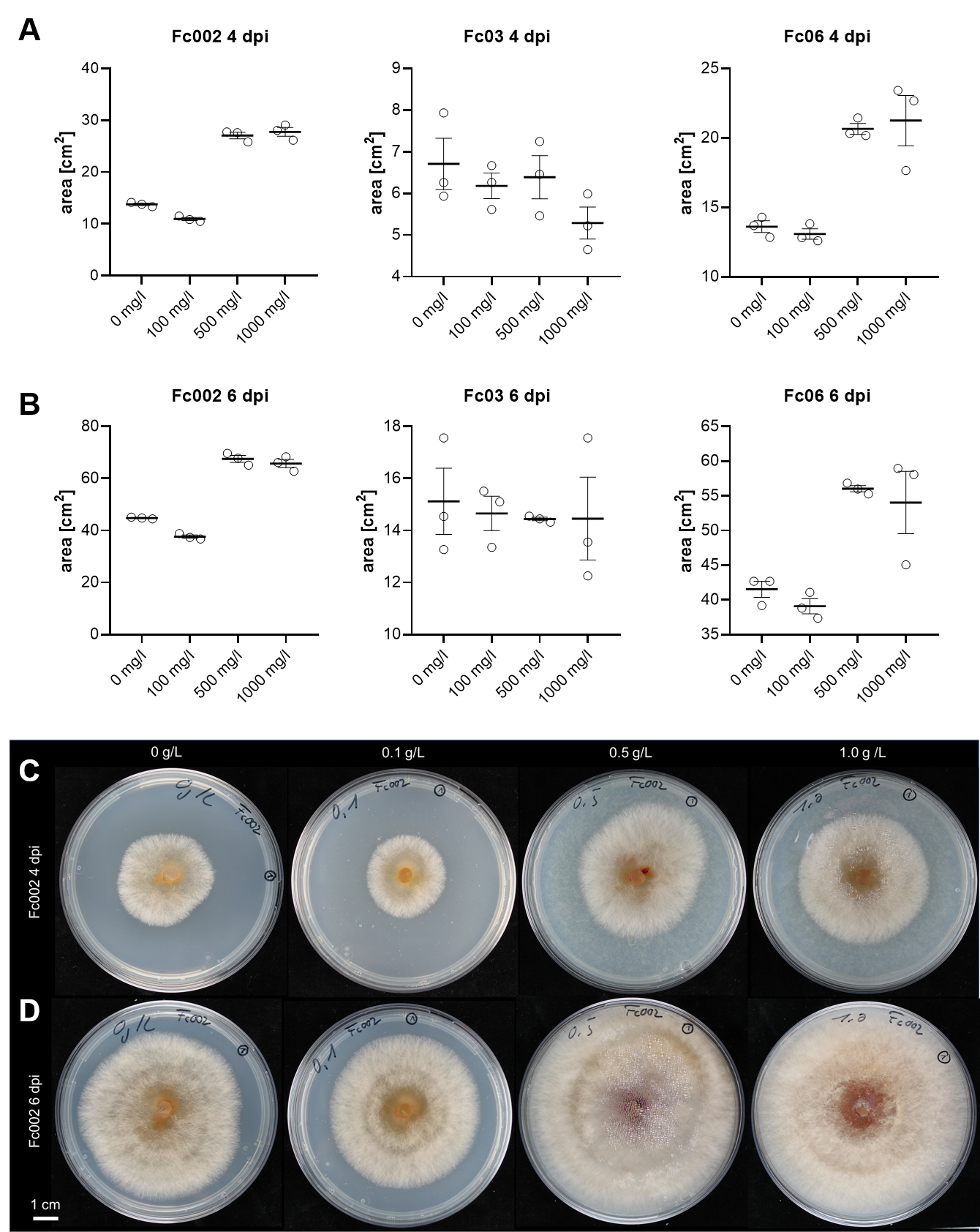


**Figure S-2: Fungal growth assay on ¼ strength PDA supplemented with different chitosan concentrations.** [A] Growth area of *F. culmorum* measured at 4 dpi. [B] Growth area of *F. culmorum* measured at 6 dpi. [C] Exemplary images of agar plates inoculated with isolate Fc002 at 4 dpi. [D] Exemplary images of agar plates inoculated with isolate Fc002 at 6 dpi. The fungal growth assay was conducted for three different *F. culmorum* isolates (Fc002, Fc03, Fc06) on ¼ strength PDA supplemented with 0.0, 0.1, 0.5 and 1.0 g L^-1^ chitosan (C3646, obtained from Sigma-Aldrich, Germany). The dot plots show mean area with fungal growth of three different agar plates per fungal isolate and chitosan concentration measured with the software ImageJ. Error bars indicate the standard error.


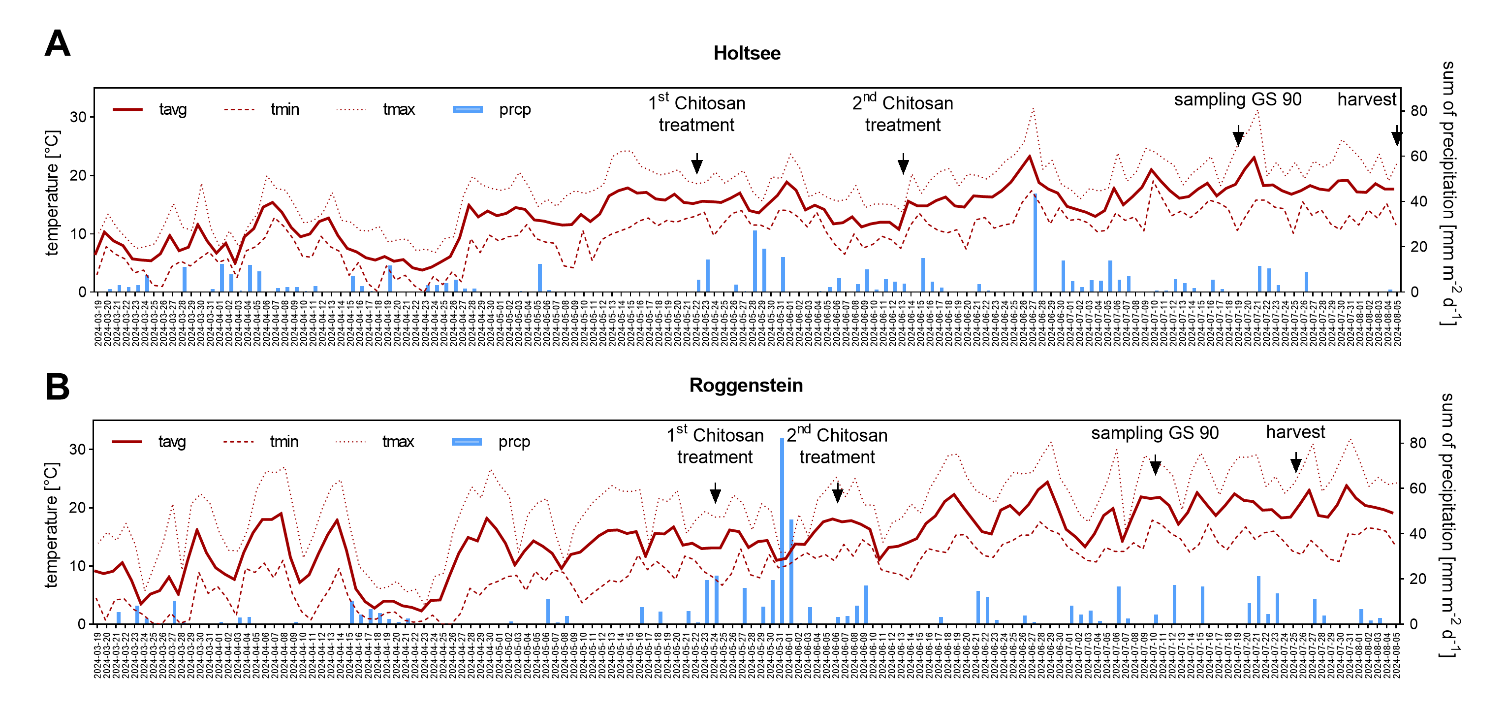


**Figure S-3: Temperature and precipitation data during the field trials at two locations in northern and southern Germany.** Daily mean (tavg), minimum (tmin), and maximum (tmax) temperatures, as well as daily precipitation sums (prcp), are shown for the period between sowing and harvest at Holtsee [A] and Roggenstein [B]. Arrows indicate the dates of the first and second chitosan treatments, grain sampling, and final harvest.


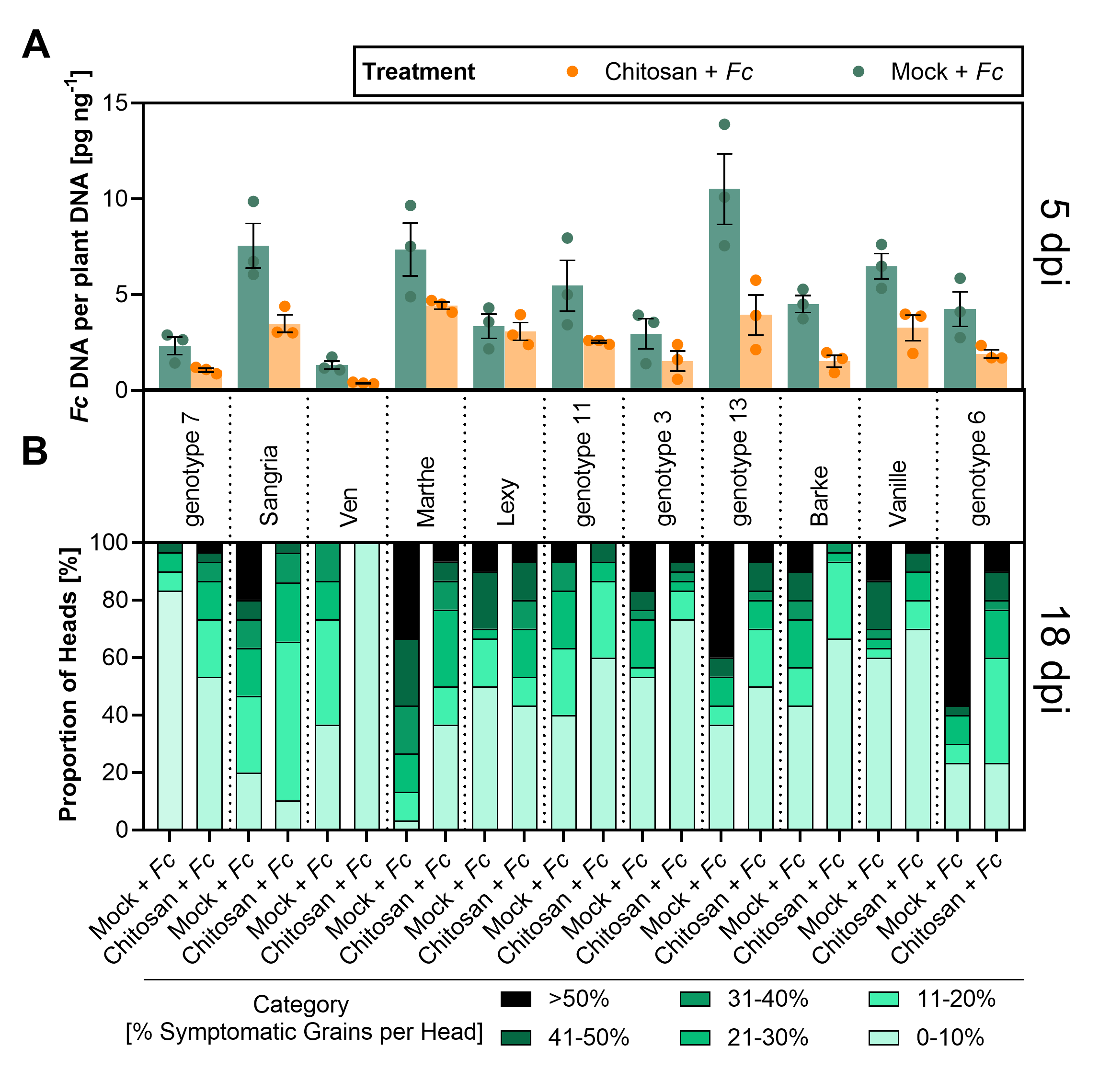


**Figure S-4: Fungal DNA contents and proportions of barley heads showing grain symptoms after mock- or chitosan-treatment and *Fusarium culmorum* infection in 11 selected barley genotypes.** [A] shows the mean *F. culmorum* DNA detected in barley heads at 5 days post inoculation. [B] displays the proportions of symptomatic grains per head are sorted by disease categories, reflecting the disease severity of individual spikes. For quantification of fungal DNA, 3 x 2 heads per sample and genotype were harvested. Grain symptoms were assessed on 30 heads per treatment group and genotype. The genotypes are sorted in the same order from left to right as displayed in Figure 2. Error bars show standard error.
